# The Impact of New Weight-Loss Medications on Bariatric Surgery and Surgeon Employment

**DOI:** 10.7759/cureus.83903

**Published:** 2025-05-11

**Authors:** Mena Louis, Eric Velazquez

**Affiliations:** 1 General Surgery, Northeast Georgia Medical Center Gainesville, Gainesville, USA; 2 Bariatric Surgery, Northside Hospital Duluth, Duluth, USA

**Keywords:** bariatric surgery, bariatric surgery jobs, glp-1 receptor agonists, multidisciplinary care, obesity, obesity management, pharmacotherapy, semaglutide, tirzepatide, weight-loss medications

## Abstract

Obesity continues to pose an urgent public health challenge in the United States, with prevalence among adults and significant associations with chronic conditions, including diabetes, cardiovascular disease, and nonalcoholic fatty liver disease. While bariatric surgery has established itself as the most effective long-term treatment for severe obesity, delivering consistent weight loss and notable improvements in obesity-related comorbidities, its utilization remains relatively low due to factors such as patient apprehension, access disparities, and insurance limitations. The recent emergence and widespread adoption of potent anti-obesity medications, particularly glucagon-like peptide-1 (GLP-1) receptor agonists like semaglutide and dual incretin agonists such as tirzepatide, have created new, non-surgical pathways capable of achieving substantial weight loss outcomes, thus prompting a reevaluation of the traditional obesity treatment landscape.

This comprehensive review assesses the contemporary state of bariatric surgery and systematically examines the clinical efficacy, safety, and durability of emerging pharmacotherapies relative to surgical interventions. We also analyze the potential implications of these medications on patient preferences, surgical volumes, healthcare economics, and the bariatric surgery job market within the United States. Recognizing the evolving role of bariatric surgeons, we discuss how the profession may adapt through multidisciplinary care models, integrated pharmacological management, and adjusted surgical training programs. The goal of this manuscript is to inform clinicians, healthcare systems, policymakers, and patients regarding the anticipated impact of novel weight-loss medications on the future direction of obesity care and the professional landscape of bariatric surgery.

## Introduction and background

Obesity has emerged as one of the most pressing global public health crises of our time, characterized by escalating prevalence rates, severe metabolic sequelae, and immense economic burdens on healthcare systems worldwide [1]. In the United States, obesity now affects more than 40% of adults and remains strongly associated with increased risks for type 2 diabetes mellitus, cardiovascular diseases, obstructive sleep apnea, chronic kidney disease, nonalcoholic fatty liver disease, and certain cancers [2]. This epidemic not only deteriorates individual quality of life but also substantially amplifies healthcare expenditures, emphasizing an urgent need for effective, scalable, and sustainable treatments [3].

Traditionally, the management of obesity has revolved around lifestyle modification strategies emphasizing dietary changes and physical activity [4]. However, for patients with severe obesity, such interventions frequently yield modest or transient results. Pharmacological therapies have historically offered supplemental benefits but have been hampered by limited efficacy, poor long-term adherence, and adverse side-effect profiles [5]. Consequently, bariatric surgery has stood out prominently as the most effective intervention for sustainable weight reduction, achieving superior outcomes in long-term weight loss, remission of diabetes, and significant improvement in quality of life metrics [6].

Despite these advantages, bariatric surgery remains underutilized, with less than 1% of eligible individuals opting for surgery each year in the United States [7]. Multiple factors contribute to this discrepancy, including patient apprehension toward invasive procedures, limited healthcare provider awareness, socioeconomic and regional access disparities, insurance coverage variability, and ongoing stigma associated with obesity and its surgical treatment [8]. As a result, a significant gap persists between available surgical care and actual treatment uptake, prompting continued exploration into less invasive yet comparably effective alternatives.

The recent development and regulatory approval of new classes of pharmacotherapies, particularly glucagon-like peptide-1 (GLP-1) receptor agonists like semaglutide and dual incretin receptor agonists such as tirzepatide, represent transformative advancements in medical weight management [9,10]. These medications have demonstrated unprecedented weight loss outcomes in clinical trials, rivaling or even approaching results previously achievable only through surgical interventions. Their availability has sparked a crucial discourse on the evolving relationship between medical and surgical obesity treatments. In addition to clinical implications, these changes carry significant professional consequences for bariatric surgeons, who may face shifting referral patterns, evolving roles in multidisciplinary care, and new expectations around medical weight-loss management. This review aims to critically analyze the emerging impact of these pharmacologic agents on bariatric surgery practices, clinical decision-making processes, healthcare economic implications, and the future employment landscape for bariatric surgeons in the United States.

## Review

History and evolution of bariatric surgery

The history of bariatric surgery traces back to the mid-20th century, evolving significantly in technique, safety, and outcomes over subsequent decades [11]. The first widely recognized bariatric operation was the jejunoileal bypass, introduced in the 1950s and 1960s, which achieved substantial weight loss through intestinal malabsorption [12]. However, significant complications of JIB, including severe diarrhea, life-threatening liver failure, and profound nutritional deficiencies, ultimately limited its clinical acceptability and prompted a moratorium on its use by the 1970s [13]. These early experiences highlighted the risks of purely malabsorptive approaches and motivated surgeons to develop safer, more effective strategies, ultimately leading to both restrictive procedures (focusing on stomach size reduction) and combined malabsorptive-restrictive procedures.

In the late 1960s and early 1970s, Dr. Edward Mason pioneered gastric bypass surgery, specifically Roux-en-Y gastric bypass (RYGB), combining restriction of stomach size and limited malabsorption [14]. Over subsequent years, the procedure underwent modifications and refinements, notably through the adoption of laparoscopic approaches in the 1990s, which drastically reduced perioperative morbidity and improved patient recovery [15]. Sleeve gastrectomy, initially performed as the first stage of a duodenal switch operation, became widely adopted as a standalone procedure in the early 2000s, offering substantial weight loss with fewer nutritional complications compared to bypass procedures [16].

The introduction of laparoscopy and, more recently, robotic-assisted surgery marked a transformative shift in bariatric surgery, making these procedures safer, more acceptable, and significantly more accessible to patients [17]. Concurrently, the establishment of national accreditation programs, such as the Metabolic and Bariatric Surgery Accreditation and Quality Improvement Program (MBSAQIP), ensured high standards of patient care, reducing surgical complications and improving long-term outcomes [18]. Today, bariatric surgery is widely accepted as the most effective long-term treatment for severe obesity, with robust evidence demonstrating sustained weight loss, improved quality of life, and substantial remission rates of obesity-associated comorbidities, including type 2 diabetes, hypertension, dyslipidemia, and sleep apnea [19].

Dr. Edward Mason pioneered gastric bypass surgery, specifically the RYGB, in the late 1960s and early 1970s, which combined restriction of stomach size with a shorter intestinal bypass to induce malabsorption [14]. This innovation marked a significant advance, producing substantial weight loss with fewer metabolic complications than the JIB. Over the subsequent decades, bariatric surgery diversified through global surgical innovation. In 1978, Nicola Scopinaro introduced the biliopancreatic diversion (BPD) in Italy as an aggressive malabsorptive procedure for severe obesity, while Mason and others developed the vertical banded gastroplasty (VBG) in the early 1980s as a purely restrictive stomach-stapling technique. By the late 1980s, the concept of adjustable gastric banding had emerged, Dr. Lubomyr Kuzmak’s early design of an inflatable silicone gastric band was later refined into the laparoscopic Lap-Band system in the early 1990s, offering a less invasive, reversible weight-loss option that gained popularity, especially in Europe and Australia [13]. Each of these new procedures aimed to improve weight loss outcomes while minimizing the serious complications seen with the JIB, reflecting an international effort to find the optimal surgical solution for obesity.

The 1990s marked a major turning point with the introduction of minimally invasive techniques. Bariatric surgeons began adopting laparoscopic approaches, initially for gastric banding and then for more complex operations, culminating in the first laparoscopic RYGB in 1994. The shift from large open incisions to laparoscopy drastically reduced surgical trauma, perioperative morbidity, and recovery times for patients, while achieving equivalent weight loss results [15]. Surgeons also explored variations of the gastric bypass itself; notably, a simplified “mini-gastric bypass” (now often termed one-anastomosis gastric bypass) was proposed in 1997 as a single-loop variant of RYGB. Although initially met with skepticism in the United States, this one-anastomosis technique gained traction in Europe and Asia as a streamlined alternative to the traditional RYGB, highlighting the global exchange of bariatric surgical ideas during this era. Entering the 2000s, sleeve gastrectomy emerged from its origin as part of the duodenal switch procedure to become a dominant standalone surgery [16]. Initially employed as the first stage of a two-step BPD/duodenal switch for high-risk patients, the sleeve gastrectomy demonstrated such effective weight loss that it was soon adopted as a primary procedure. By removing roughly 80% of the stomach along the greater curvature, the sleeve offered substantial weight reduction with a lower risk of micronutrient deficiencies and surgical complexity compared to gastric bypass [16]. By the mid-2010s, sleeve gastrectomy had in fact become the most commonly performed bariatric procedure worldwide, reflecting its favorable balance of efficacy and safety in the eyes of surgeons and patients.

The introduction of advanced minimally invasive technology further transformed the field in the 2000s and 2010s. The adoption of robotic-assisted bariatric surgery built upon the laparoscopic revolution by enhancing surgical precision and ergonomics for the surgeon. Robotic systems (e.g., the da Vinci Surgical System, Intuitive Surgical, Inc., CA, USA) were applied to procedures such as RYGB and sleeve gastrectomy, facilitating fine dissection and complex suturing with wristed instruments and 3D visualization. While high costs and learning curves initially limited the spread of robotics, this approach has made certain challenging operations (such as revisional bariatric surgeries) more feasible and consistent, and its utilization has grown steadily in high-volume centers [17]. Collectively, the widespread adoption of laparoscopy and the introduction of robotics marked a transformative shift in bariatric surgery, dramatically improving perioperative safety and making surgical weight loss more acceptable and accessible to patients than ever before [17]. In parallel with operative innovations, the field has expanded into less invasive endoscopic bariatric therapies in recent years. Techniques such as endoscopic sleeve gastroplasty and intragastric balloon placement have emerged as adjunctive options that require no incisions. These endoluminal procedures can often be performed on an outpatient basis and avoid the risks of general surgery, thereby appealing to a broader range of patients, although the magnitude of weight loss achieved tends to be less than that of operative procedures [19]. The evolution of endoscopic approaches highlights the ongoing drive to improve the risk-benefit profile of obesity treatment and fill the gap for patients who may not qualify for or desire standard surgery.

Concurrently, major efforts were undertaken to standardize care and improve outcomes as bariatric surgery entered the mainstream. Professional organizations and health systems recognized that rigorous standards and accreditation were essential to ensuring safety as more hospitals and surgeons began offering these operations. In the United States, the American Society for Metabolic and Bariatric Surgery (ASMBS) and the American College of Surgeons collaboratively established national accreditation programs for bariatric centers of excellence in the 2000s, culminating in the unified MBSAQIP by 2012. These accreditation programs set minimum volume requirements, protocols for multidisciplinary care, and data reporting mandates that collectively ensured high standards of patient care. The result was a measurable reduction in surgical complications and improvements in long-term outcomes at accredited centers, helping to standardize quality across the country [18]. Similar accreditation and training initiatives have been promoted internationally (for example, through the International Federation for the Surgery of Obesity and Metabolic Disorders, founded in 1995), contributing to more consistent best practices worldwide [18].

Today, bariatric surgery has evolved from an experimental, high-risk intervention into a well-established, standardized treatment modality available across the globe. It is widely accepted as the most effective long-term therapy for severe obesity, with robust evidence demonstrating sustained weight loss, improved quality of life, and high remission rates of obesity-associated comorbidities (including type 2 diabetes, hypertension, dyslipidemia, and sleep apnea) [19]. Large-scale studies have documented not only these metabolic and quality-of-life benefits but also significant reductions in obesity-related mortality among post-bariatric patients, underlining the profound impact of surgery on long-term health [19]. The journey of bariatric surgery over the past several decades, from the crude malabsorptive bypasses of the 1950s to the sophisticated, multi-modal, and team-based care of the present day, epitomizes remarkable progress in surgical innovation, patient safety, and clinical outcomes. This historical evolution has established bariatric surgery as a cornerstone of modern obesity management, continually adapting with emerging technologies and adjunctive therapies to further enhance safety, access, and effectiveness for patients worldwide.

History and evolution of weight loss medications

**Figure 1:**
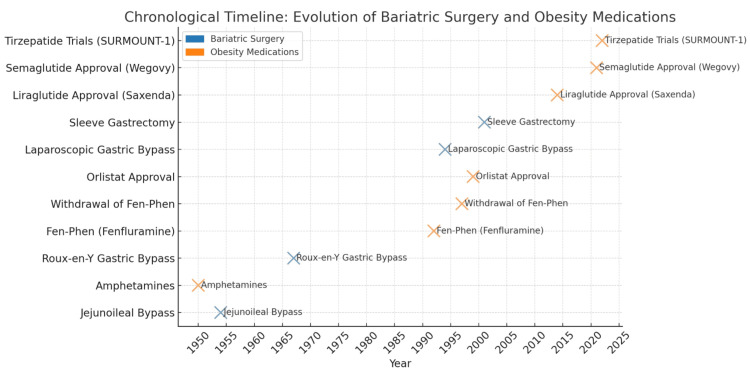
Chronological timeline clearly illustrating the historical evolution of bariatric procedures alongside key milestones in obesity medications. Data compiled from multiple sources, including [23], as well as published outcomes from the Surgical Treatment and Medications Potentially Eradicate Diabetes Efficiently (STAMPEDE) trial, Semaglutide Treatment Effect in People with obesity (STEP) 1 and 2 trials, SURMOUNT-1, and long-term follow-up data from bariatric surgery studies.

Pharmacological treatments for obesity have traversed a turbulent pathway since the mid-20th century (Figure 1), characterized by alternating periods of high enthusiasm and abrupt withdrawals due to safety concerns or efficacy limitations [20]. Early drug therapies were primarily aimed at appetite suppression through central nervous system stimulation, exemplified by amphetamines in the 1950s and 1960s [21]. Amphetamines did indeed promote weight reduction; however, their propensity to cause addiction, tachycardia, and psychiatric disturbances led to stringent regulatory measures and a marked decline in use [22]. This initial setback foreshadowed the delicate balance of risks and benefits inherent in many anti-obesity agents, driving researchers and regulatory bodies to scrutinize subsequent pharmacotherapies with heightened caution.

The 1990s witnessed the rise and fall of fenfluramine and dexfenfluramine, frequently prescribed in conjunction with phentermine as the widely known “fen-phen” combination [24]. By manipulating serotonin reuptake and inducing satiety, fen-phen gained rapid popularity as a seemingly revolutionary weight-loss strategy. However, severe adverse events, specifically cardiac valvulopathy and pulmonary hypertension, sounded a critical alarm, culminating in market withdrawal by 1997 [25]. In the fallout from fen-phen, regulatory agencies worldwide adopted more rigorous safety protocols, prompting the pharmaceutical industry to seek novel mechanisms of action. Around this period, orlistat emerged as a non-central nervous system alternative that inhibited pancreatic and gastric lipases, thereby reducing fat absorption [26]. Although orlistat produced moderate weight loss and demonstrated a tolerable safety profile compared to amphetamine-based agents, side effects like steatorrhea and fecal urgency continued to hamper long-term patient adherence.

Global markets continued to explore additional medications to fill the post-fen-phen gap. Some agents acted on various neurotransmitter pathways, while others sought to modulate satiety via gut hormone receptors. Lorcaserin, for instance, briefly generated optimism by selectively targeting serotonin 2C receptors, aiming to minimize cardiac side effects observed in fenfluramine [27]. Despite initial approval, lorcaserin faced eventual withdrawal due to concerns about potential cancer risks, which illustrates the complexities of finding pharmacotherapies that remain both efficacious and consistently safe over extended use. In parallel, countries outside of the United States, especially in Europe and parts of Asia, experimented with other novel appetite suppressants or sympathomimetic agents, though few gained widespread acceptance due to similar safety or efficacy issues.

**Figure 2:**
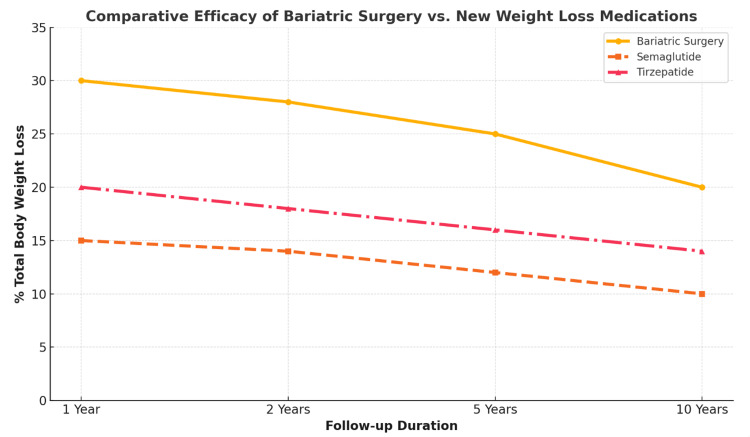
A comparative efficacy graph clearly illustrating the percentage of total body weight loss achieved through bariatric surgery versus new weight-loss medications (semaglutide and tirzepatide) at different follow-up durations (one, two, five, and 10 years). Data compiled from multiple sources, including [23], as well as published outcomes from the Surgical Treatment and Medications Potentially Eradicate Diabetes Efficiently (STAMPEDE) trial, Semaglutide Treatment Effect in People with obesity (STEP) 1 and 2 trials, SURMOUNT-1, and long-term follow-up data from bariatric surgery studies.

Over the past decade, a pivotal shift has occurred in obesity pharmacotherapy, gravitating away from generalized appetite suppressants toward agents with more targeted hormonal and metabolic effects [28]. GLP-1 receptor agonists, originally approved for the management of type 2 diabetes, emerged as promising anti-obesity medications after demonstrating significant weight reduction alongside improved glycemic control. Semaglutide and liraglutide are notable members of this class, achieving average weight losses that notably surpass earlier oral agents. Their mechanisms of delaying gastric emptying, reducing appetite through central satiety signals, and improving insulin sensitivity reflect a more nuanced approach than previous stimulant-based drugs [9,10]. The introduction of dual-incretin agonists, such as tirzepatide, further amplified these outcomes, inching closer to weight-loss efficacy that begins to parallel bariatric surgery (Figure 2).

**Figure 3:**
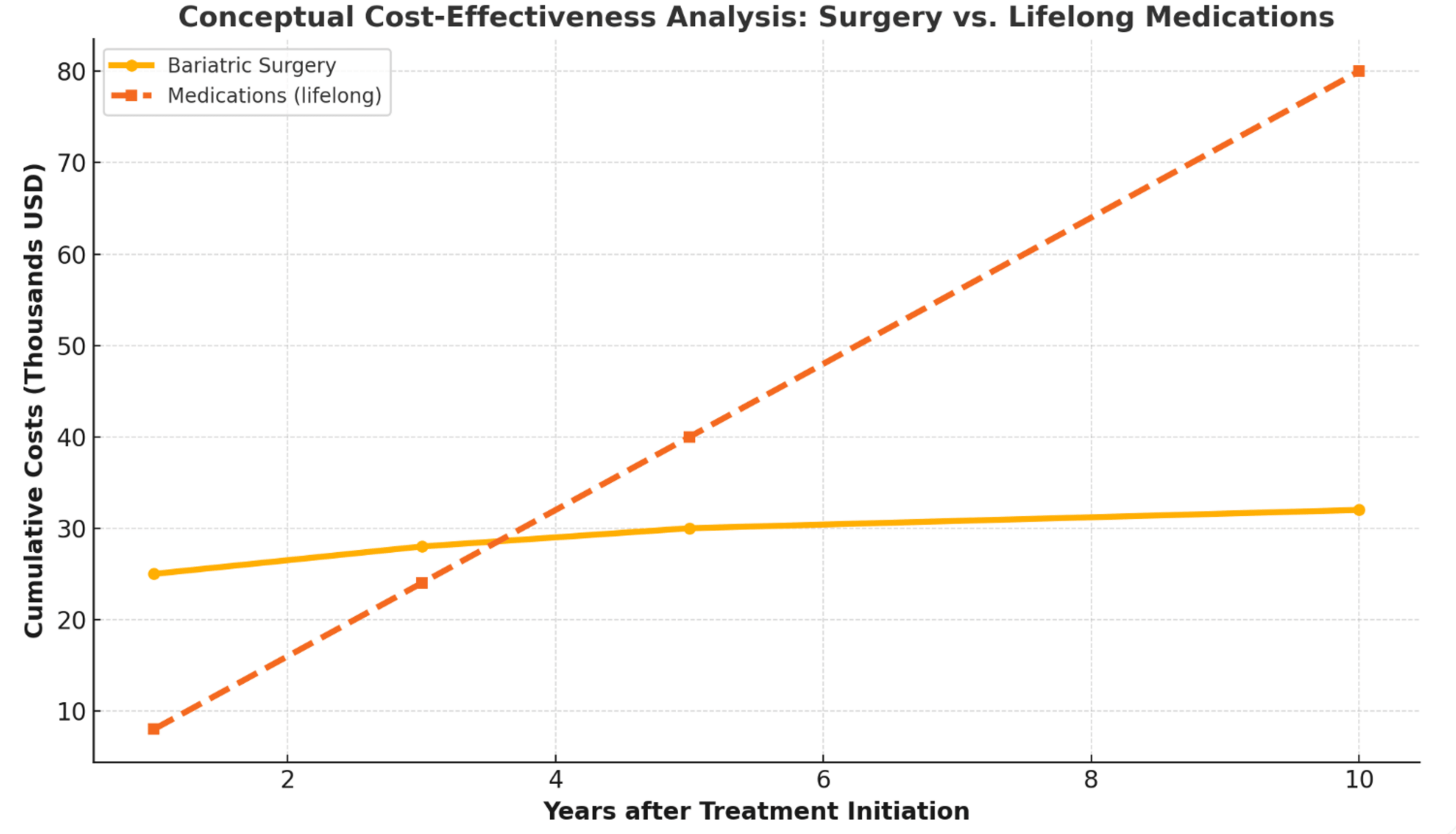
Conceptual cost-effectiveness analysis comparing the cumulative costs of bariatric surgery versus lifelong medication-based weight management. Although bariatric surgery involves higher upfront costs, cumulative long-term costs stabilize over time. In contrast, pharmacological treatment starts with lower initial costs but escalates substantially with continuous lifelong therapy. Data compiled from multiple sources, including [23], as well as published outcomes from the Surgical Treatment and Medications Potentially Eradicate Diabetes Efficiently (STAMPEDE) trial, Semaglutide Treatment Effect in People with obesity (STEP) 1 and 2 trials, SURMOUNT-1, and long-term follow-up data from bariatric surgery studies.

These advances in pharmacotherapy have sparked a paradigm shift in obesity management, wherein high-efficacy medications can serve as viable alternatives or adjuncts to surgery. Importantly, the improved safety profile of these agents, particularly concerning cardiovascular risks, has fueled greater acceptance among clinicians and patients. Although questions persist about long-term adherence, cost, and durability of weight reduction once treatment ceases, these new drugs have indisputably altered the therapeutic landscape (Figure 3). They hold the potential to reshape patient preferences and clinical algorithms, positioning pharmacotherapy not only as a stepping stone toward surgery for some but also as a standalone option for many who either do not qualify for or are reluctant to undergo surgical intervention. In this evolving environment, it remains to be seen how the continued refinement of these medications will further influence the interplay between medical and surgical obesity treatments, potentially redefining the standard of care for years to come.

Methods

This narrative review was conducted by searching PubMed, Google Scholar, and Scopus for English-language articles published from 2000 to 2024. Search terms included combinations of “bariatric surgery,” “GLP-1 receptor agonists,” “semaglutide,” “tirzepatide,” “anti-obesity medications,” and “obesity management.” We included clinical trials, meta-analyses, reviews, and relevant opinion pieces that addressed the efficacy, safety, economic impact, and professional implications of both surgical and pharmacologic obesity treatments. Studies focusing exclusively on pediatric populations or non-obesity-related indications were excluded. Priority was given to high-quality, peer-reviewed publications and recent landmark studies. A flowchart summarizing the literature selection process is provided for reference.

Discussion

The emergence of novel pharmacological treatments, particularly GLP-1 receptor agonists such as semaglutide and dual incretin receptor agonists like tirzepatide, represents one of the most significant developments in obesity management in recent decades [29]. These agents have demonstrated efficacy levels that, until recently, were considered attainable only through surgical interventions. Clinical trials consistently show that these medications can lower body weight by 15-20%, a remarkable shift that challenges formerly held assumptions about the limitations of non-surgical weight loss [30]. Although this pharmacological progress offers a less invasive strategy for treating obesity, the implications for established bariatric procedures, as well as for overall healthcare resource allocation, demand thorough examination [31].

Historically, bariatric surgery has proven to be the most reliable method of achieving both significant and sustained weight reduction among severely obese patients [12]. Key procedures, most notably RYGB and sleeve gastrectomy, yield consistent improvement in metabolic comorbidities, particularly type 2 diabetes, which often achieves remission shortly after surgery. Long-term evidence, including data from landmark investigations such as the Swedish Obese Subjects (SOS) study and the Surgical Treatment and Medications Potentially Eradicate Diabetes Efficiently (STAMPEDE) trial, highlights the superior durability of surgical outcomes for both weight loss and cardiometabolic risk mitigation [32,33]. Despite this compelling evidence, bariatric surgery remains underutilized, largely due to patient apprehensions regarding invasive procedures, misconceptions about surgical risks, lack of awareness among certain healthcare providers, and the persisting stigma surrounding obesity [8]. Moreover, insurance coverage limitations and socioeconomic barriers exacerbate this gap, leaving a considerable proportion of eligible patients untreated [3]. Effective pharmacotherapies may now fill a portion of this space; however, the extent to which they can replicate the comprehensive metabolic benefits and the high remission rates of obesity-related diseases offered by surgery is not yet fully delineated.

The development of semaglutide and tirzepatide has unquestionably altered clinical treatment algorithms [9,10]. Semaglutide Treatment Effect in People with obesity (STEP) trials demonstrated average weight losses of about 15% over two years, while tirzepatide’s SURMOUNT-1 trial pushed these figures to nearly 20% for certain dose regimens [34]. Nevertheless, uncertainties remain about the long-term sustainability of these pharmacologically induced outcomes when patients discontinue medication and about the capacity of these drugs to address severe comorbidities as comprehensively as surgery [30]. A key question pertains to weight regain upon cessation of therapy: while surgery physically and hormonally curbs the potential for rapid relapse, pharmacotherapy relies on consistent patient adherence and continued health coverage for an extended duration [35]. This scenario shows the multifaceted challenges around medication costs, insurance policies, side-effect management, and patient compliance.

Clinically, the advent of these potent medications may particularly influence treatment choices among patients with BMIs between 30 and 40 who are borderline candidates for surgery or are reluctant to undergo an operative procedure [3]. For this subset of patients, non-surgical methods, now that they can achieve double-digit percentages in weight loss, offer a compelling alternative that may reduce bariatric surgical volume. Nevertheless, for individuals with severe obesity (BMI ≥40) or extensive obesity-related comorbidities, bariatric surgery likely will remain the leading therapeutic modality by virtue of its enduring metabolic impact and ability to induce profound, sustained weight reduction [19]. Of note, pharmacotherapy may become an increasingly integral component of multidisciplinary programs, serving as a preoperative strategy to mitigate surgical risk by achieving initial weight loss or as a postoperative adjunct to maintain weight stability and enhance long-term outcomes [36]. Such integrated models of care may forge more comprehensive, patient-tailored treatment pathways.

The emergence of highly effective anti-obesity medications has not only transformed treatment options but also altered the professional landscape for bariatric surgeons. As more patients pursue non-surgical therapies, particularly those with lower BMI ranges who may have previously been candidates for surgery, there is growing concern about reduced surgical volume and the long-term implications for surgical practice. However, this shift also presents an opportunity for surgeons to redefine their role and remain at the forefront of obesity care.

Surgeons who expand their practices to include medical weight-loss management, pharmacotherapy, and minimally invasive endoscopic procedures can serve a broader population [28]. This diversification allows them to guide patients through all stages of obesity management, from initial counseling and pharmacologic interventions to operative care when appropriate. Rather than being limited to procedural roles, bariatric surgeons can evolve into comprehensive metabolic health providers, playing a central part in multidisciplinary care teams. This transition may also support long-term career sustainability in the face of changing referral patterns. Surgeons who engage in medical weight-loss consultations, participate in collaborative care models, and contribute to longitudinal obesity management are well-positioned to adapt. As the field continues to evolve, the ability to integrate both surgical and medical therapies will likely become essential to maintaining a robust and relevant practice.

Economic dimensions further complicate this evolving landscape [22]. Although pharmacotherapy is often perceived as more cost-effective than surgery initially, the requirement for lifelong medication in many cases can accrue substantial costs over extended durations, potentially surpassing the single, more pronounced expense of a bariatric procedure [30]. Additionally, the success of medical therapies hinges on adequate insurance coverage, which may vary significantly by region or plan. Because economic factors inherently affect patient access to both surgery and medications, the potential shift away from operative interventions has implications for health policy, hospital resource allocation, and the bariatric job market. Policymakers and payers must weigh the upfront costs of surgery, along with its associated improvement in obesity-related conditions, against the cumulative expenses and potential nonadherence tied to chronic medication use. Cost-effectiveness analyses that account for reductions in hospital admissions, improvements in quality of life, and productivity gains related to weight reduction become pivotal to guide future reimbursement structures.

In response, bariatric surgeons would benefit from adopting a multifaceted skill set that includes prescribing or at least partnering closely with specialists to manage advanced pharmacotherapies. Such an expansion of capabilities does not negate the importance of surgical expertise but rather complements it, creating a continuum of options for individuals across different obesity severities, comorbidities, and personal preferences. Incorporating these pharmacological agents can appeal to patients who are ambivalent about surgery, potentially increasing the overall pool of individuals engaging with weight-loss treatment, while still maintaining surgical solutions for those who need or prefer them. Furthermore, surgeons who actively participate in clinical trials or post-market studies of these medications can stay at the cutting edge of evolving evidence, clarify optimal use cases, and ensure that patient selection protocols remain meticulous and personalized.

Professional organizations, such as the ASMBS, can facilitate these transitions by offering specialized training modules, workshops, and certification pathways. Surgeons trained in both operative and medical obesity management can offer more holistic patient evaluations and robust long-term follow-up, incorporating medication adjustments, nutrition counseling, and psychosocial support. This interdisciplinary synergy has the potential to improve patient satisfaction, reduce readmissions for weight regain, and enhance overall treatment outcomes for severe obesity.

Ultimately, integrating potent pharmacotherapy and bariatric surgery not only diversifies the strategies available to patients but also incentivizes the healthcare system to pursue combined approaches that yield optimal metabolic and quality-of-life outcomes. Whether a particular patient pursues a purely medical strategy, a purely surgical one, or a hybrid approach will depend on factors such as BMI, comorbidities, cost, insurance coverage, readiness for surgery, and personal preference. As our understanding of obesity’s heterogeneous pathophysiology advances, more personalized treatment algorithms will likely emerge, placing bariatric surgeons at the forefront of precision obesity management.

The recent surge in effective weight-loss medications marks a pivotal moment in obesity care, presenting opportunities to address patient populations previously unreached by surgery alone. While the longevity and breadth of these medications’ metabolic benefits remain subject to further evaluation, they are clearly reshaping clinical practice. Rather than signaling a diminishing role for bariatric surgeons, this new treatment environment may strengthen the profession’s adaptability and relevance. Surgeons who proactively expand their skill sets and engage in multidisciplinary collaboration are well-positioned to continue leading the fight against obesity in both operative and non-operative domains.

## Conclusions

The development and widespread adoption of highly effective pharmacological treatments for obesity, particularly GLP-1 receptor agonists and dual incretin agonists, signify a substantial shift in the landscape of obesity care. While these medications present promising non-surgical weight-loss alternatives, bariatric surgery remains indispensable, especially for patients requiring significant and sustained metabolic improvements. Bariatric surgeons should adapt by integrating pharmacotherapy into their practice, enhancing multidisciplinary collaboration, and advocating for equitable access to comprehensive obesity treatments. Ultimately, a personalized approach combining both medical and surgical interventions will yield optimal patient outcomes and shape the future of obesity management.
